# Genetic Structure and Eco-Geographical Differentiation of Wild Sheep Fescue (*Festuca ovina* L.) in Xinjiang, Northwest China

**DOI:** 10.3390/molecules22081316

**Published:** 2017-08-09

**Authors:** Chenglin Zhang, Jianbo Zhang, Yan Fan, Ming Sun, Wendan Wu, Wenda Zhao, Xiaopeng Yang, Linkai Huang, Yan Peng, Xiao Ma, Xinquan Zhang

**Affiliations:** 1 Department of Grassland Science, Animal Science and Technology College, Sichuan Agricultural University, Chengdu 611130, China; zcl0636@126.com (C.Z.); sunming4709@163.com (M.S.); wuwendandan@163.com (W.W.); zwd89897@163.com (W.Z.); 18227587409@163.com (X.Y.); huanglinkai@sicau.edu.cn (L.H.); pengyanlee@163.com (Y.P.); 2 Sichuan Academy of Grassland Science, Chengdu 610097, China; zhangjianber@163.com; 3 Chongqing Academy of Animal Husbandry, Chognqing 400039, China; cq_fy001@163.com

**Keywords:** *Festuca ovina* L., AFLP, genetic diversity, genetic barriers, environmental factors

## Abstract

Glaciation and mountain orogeny have generated new ecologic opportunities for plants, favoring an increase in the speciation rate. Moreover, they also act as corridors or barriers for plant lineages and populations. High genetic diversity ensures that species are able to survive and adapt. Gene flow is one of the most important determinants of the genetic diversity and structure of out-crossed species, and it is easily affected by biotic and abiotic factors. The aim of this study was to characterize the genetic diversity and structure of an alpine species, *Festuca ovina* L., in Xinjiang, China. A total of 100 individuals from 10 populations were analyzed using six amplified fragment length polymorphism (AFLP) primer pairs. A total of 583 clear bands were generated, of which 392 were polymorphic; thus, the percentage of polymorphic bands (PPB) was 67.24%. The total and average genetic diversities were 0.2722 and 0.2006 (0.1686–0.2225), respectively. The unweighted group method with arithmetic mean (UPGMA) tree, principal coordinates analysis (PCoA) and Structure analyses revealed that these populations or individuals could be clustered into two groups. The analysis of molecular variance analysis (AMOVA) suggested that most of the genetic variance existed within a population, and the genetic differentiation (Fst) among populations was 20.71%. The Shannon differentiation coefficient (G’st) among populations was 0.2350. Limited gene flow (Nm = 0.9571) was detected across all sampling sites. The Fst and Nm presented at different levels under the genetic barriers due to fragmentation. The population genetic diversity was significant relative to environmental factors such as temperature, altitude and precipitation.

## 1. Introduction

Glaciation and mountain orogeny are the main factors that shape landscapes and change climate, and they have been linked to recent diversification and speciation events [1,2]. They could create many different environments and microclimates, which provide new habitats for plants and trigger evolutionary processes [3]. Accumulating evidence suggests that historical glacial and orogenic movements, including some recent biotic activities, might cause habitat fragmentation, which seriously increases the vulnerability of many plant species and threatens their survival [4]. These factors could also alter the abundance and behavior of pollinators and restrict seed dispersal [5,6]. The primary changes during habitat fragmentation include reduced population sizes and increased spatial isolation among populations [7]. Consequently, a deleterious erosion of genetic diversity and intensification of inter-population divergence would occur by random genetic drift, elevated inbreeding, and decreased inter-population gene flow [8,9]. As the feedback, fitness, richness and adaptation ability of a population would be weakened, such changes could even cause the local extinction of demes within a meta-population [10,11].

High genetic diversity is fundamental for the ability of a species to survive and develop [12]. It is often associated with traits that enable a species to adapt, such as expanding their distribution range and creating a new niche [13,14]. Fragmentation can affect plant species differently depending on their life history traits. The genetic erosion derived from habitat fragmentation depends on multiple life traits, including population size, distance between populations, time since isolation, seed and pollen dispersal distance, and generation time [15]. For out-crossed species, gene flow via pollen and seed dispersal is one of the most important determinants in the establishment of population genetic diversity and structure [16]. However, gene flow is often interrupted by isolation and environmental heterogeneity [17,18,19]. Notably, historical climatic oscillations have greatly influenced the biodiversity of plant species. These effects were normally manifested by population contractions and expansions, long-distance range dispersal, new habitat colonization and in situ survival in glacial refugia [20,21]. Consequently, genetic erosion has occurred in some species. Researching the contemporary genetic diversity of species is required to understand ecological adaption processes and historical evolution. Hence, rational utilization and conservation practices are more effectively and efficiently realized for plant germplasms with agricultural importance. 

The genus *Festuca* (Poaceae), with its approximately 100 species, is one of the most prevalently used grass genera, presenting a wide cosmopolitan distribution in the world [22]. Among these species, *Festuca ovina* L. is a perennial, cool-season and outbreeding grass aggregate with considerable agricultural and ecological importance [23,24]. Its main ploidy levels are documented: diploid 2*n* = 4*x* = 14 and tetraploid 2*n* = 4*x* = 28 [25]. Due to its strong tolerance to stress and adversity, *Festuca ovina* L. survives well in various habitats, which range from acid soils, coastal dunes and cliffs to cold deserts and heavy metal soils. Moreover, this species is abundant in Eurasia, ranges from the Arctic to the warm temperate zone, and is even found in outlying areas further south [25,26]. *Festuca ovina* L. is often used as a constructive species, turf grass, hay or grazing grass due to its multi-tillers and nutritional value (i.e., sugar and protein content), and it also presents considerable ecological resistance to sandstorms and soil erosion in some alpine and desert areas [23,26]. Generally, *F. ovina* and *Stipa purpurea* are the dominate species of Temperate steppe in Xinjiang, China, while a large number of *Leymus*, *Potentilla* and deleterious Leguminosae plants have invaded, expanded and occupied the dominant position in degraded ecosystem because of global climate changes, overexploitation, overgrazing and pollution [27,28]. The stability and quantitative characteristics of the *F. ovina* community indicate high volatility and vulnerability and the population size is gradually decreasing, causing genetic erosion that seriously impedes the sustainable development of the region’s ecological security and local animal husbandry.

The Tianshan Mountains in Xinjiang of China and Central Asia are three parallel fold mountains that present a typical case of glaciation and orogenic movement [29,30]. They have contributed an important influence on plant biodiversity since their uplift during the early Holocene because they have acted as an East–West corridor, allowing lineage exchange, but also as a North–South barrier, promoting vicariance [31]. Moreover, desert expansion, environmental aridification, and river course dynamics have significant roles in providing adequate habitats for persistence of many plant species that could tolerate extreme drought and cold through glacial cycles [32,33]. Investigations of the genetic diversity of many species have been carried out in Xinjiang, but rarely for *F. ovina.* Actually, the heterogeneity of landscapes triggered by orogenic movements has formed many special habitats [34,35]. Many alleles and traits related to adaptation have been filtered under these special eco-geographic habitats due to their independent development at different elevations or under different edaphic characteristics, temperatures or levels of precipitation [18,36]. The morphological assessment and isozyme methods need a long time and considerable effort to establish the individuals and populations in replicated field experiments in homogenous environments, while the molecular marker techniques such as amplified fragment-length polymorphism (AFLP) analysis are more efficient because they provide much more detailed insight into genetic diversity and variation at the DNA level [37,38]. It is possible to evaluate a large number of polymorphic loci for any origin or level of DNA complexity without prior genomic information. Thus, AFLP has been widely used to determine phylogenetic history, answer important ecological questions and assess germplasm resources [39,40].

In this study, AFLP was used to investigate the genetic diversity and structural patterns of 100 wild *F. ovina* individuals from 10 populations found in Xinjiang. The aims were to (1) characterize the level of genetic diversity and the distribution of genetic variation within and among *F. ovina* populations; (2) calculate the gene flow and differentiation among populations at different scale and fragmented patterns; and (3) discuss the influence of environmental factors on population genetic diversity and structure. As a consequence, this study supplies a basis for the collection and protection of germplasm resources.

## 2. Results

### 2.1. AFLP Polymorphism and Genetic Diversity

In this study, the six selected AFLP primer pairs generated 583 clear bands that corresponded to an average of 97.17 per primer pair (Table 1). Among these bands, 392 were polymorphic. As the polymorphic bands for each primer ranged from 56 to 70, the percentage of polymorphic bands (PPB) was 67.24%. The polymorphism information content (PIC) varied from 0.1917 (E85M57) to 0.2221 (E42M85), with an average of 0.2107. The total Nei’s genetic diversity (Hj) of the primer pairs was 0.3549 (0.2036–0.3024), which corresponded to an average of 0.2531. The Shannon diversity index (Ho) of primer pairs varied from 0.20462 to 0.4406, and the total and average Ho values were 0.4642 and 0.3164, respectively.

The genetic diversities of 10 *F. ovina* populations are listed in Table 2. The number of polymorphic loci (Np) ranged from 198 (FO-04) to 294 (FO-06), and the total and average Np were 392 and 298.5, respectively. The average percentage of polymorphic loci (PPL) was 67.24% (50.5%–75.0%). The total Hj and total Shannon diversity index (Ho) were 0.2622 and 0.2988, respectively; these values were highest in FO-07 (Hj = 0.2225, Ho = 0.2505) and lowest in FO-04 (Hj = 0.1686, Ho = 0.1966). The average observed number of alleles per locus (Na) was 1.6314 (1.5026–1.7092), and the average effective number of alleles per locus (Ne) was 1.3043 (1.2585–1.3425).

### 2.2. Genetic Distance and Structure

A total of 392 fragments from six AFLP loci were used to estimate pairwise Nei’s genetic distances (GD) among 10 *F. ovina* populations (Table 3). The genetic distance was relatively low, varying from 0.0802 (FO-03 vs. FO-04) to 0.1508 (FO-05 vs. FO-09) with an average value of 0.1094. Lower genetic distances indicated a closer relationship among the studied populations, although some visible differences obviously exist between them.

**Figure 1:**
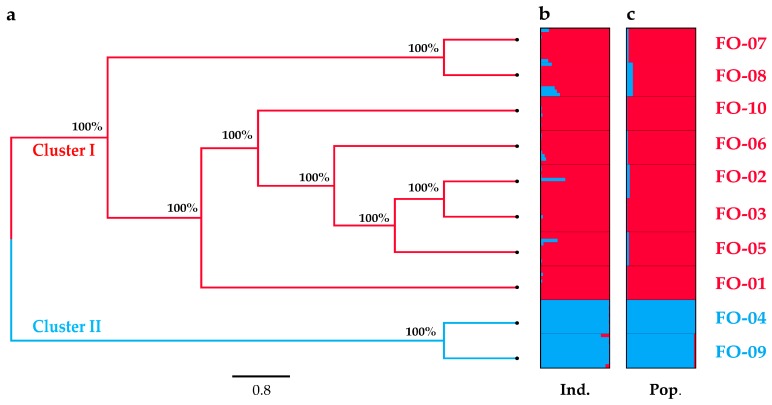
Cluster analysis for *F. ovina* ((**a**) UPGMA (unweighted pair-group method with arithmetic means) tree for 10 populations, (**b**) structure analysis for 100 individuals, (**c**) structure analysis for 10 populations). Two colors represented different potential genetic backgrounds.

To study the genetic structure of *F. ovina*, a UPGMA (unweighted pair-group method with arithmetic means) clustering analysis at the species level was conducted (Figure 1a). The results showed that the 10 populations gathered into two clusters with a bootstrapping value of 100%. Cluster I contained 8 populations (FO-01, FO-02, FO-03, FO-05, FO-06, FO-07, FO-08 and FO-10), whereas Cluster II contained 2 populations (FO-04 and FO-09). We performed a Structure analysis to further study the genetic structure (Figure 1b,c). This analysis revealed that the 10 populations clustered into two groups, FO-04 and FO-09 clustered into the same group, and the remaining 8 populations were clustered into an alternative group. This result was highly consistent with that of the UPGMA tree.

**Figure 2:**
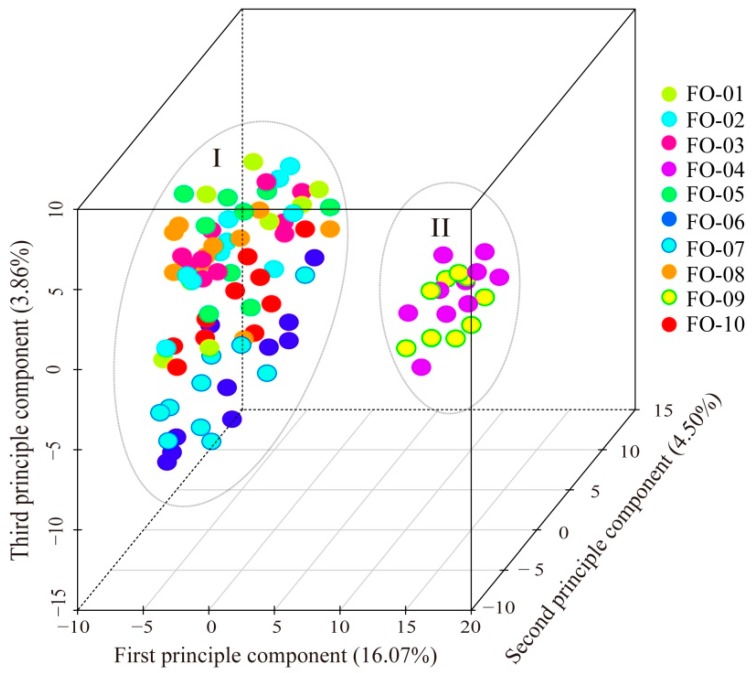
Principal coordinates analysis (PCoA) of 100 *F. ovina* individuals from 10 populations based on genetic distance matrix. The 10 individuals of per population were represented by the same dots.

Principal coordinates analysis (PCoA) was performed on the entire dataset of 100 individuals. The results showed that these individuals were divided into two groups (Figure 2). The individuals of FO-01, FO-02, FO-03, FO-05, FO-06, FO-07, FO-08 and FO-10 were gathered into Group I, whereas the individuals of FO-04 and FO-09 were gathered into Group II. The first principal vector explained 16.07% of the genetic variation, whereas the second and third principal vectors explained 4.50% and 3.86% of the genetic variation, respectively. This result was also consistent with those of the UPGMA tree and STRUCTURE analysis.

### 2.3. Genetic Differentiation, Gene Flow and Genetic Barrier

Analysis of molecular variance (AMOVA) was conducted to further evaluate the partitioning of genetic differentiation among and within *F. ovina* populations (Table 4). The results showed that 20.71% of the genetic differentiation occurred among populations, whereas 77.83% was attributable to variability within populations. Furthermore, AMOVA was conducted for the two clusters determined by the UPGMA tree and STRUCTURE analysis. The results suggested that 11.54% of the genetic differentiation occurred between the two clusters. Moreover, the Shannon differentiation coefficient was also calculated as G’st = 0.2350 among all populations. The gene flow was Nm = 0.9571 across all sampling sites.

**Figure 3:**
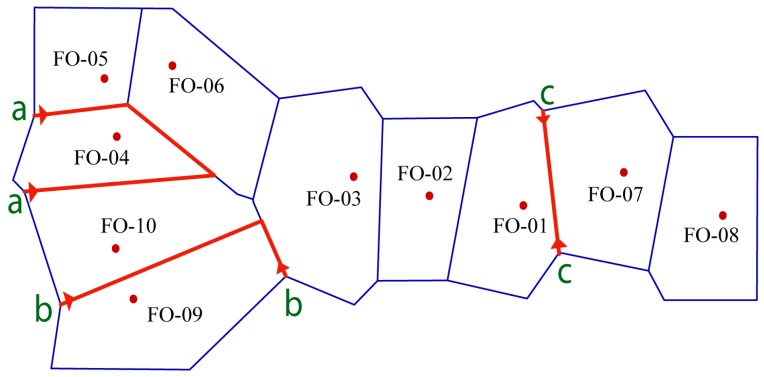
Genetic barriers predicted by BARRIER software (version 2.2, Syracuse University, New York, NY, USA). Lines a, b, and c indicated genetic barriers.

Furthermore, a genetic barrier prediction analysis for 10 *F. ovina* populations was carried out using Monmonier’s maximum difference algorithm. This analysis revealed three likely barriers against gene flow (Figure 3). The first barrier (aa) isolated FO-04 from its surrounding populations. The second barrier (bb) separated FO-09 from neighboring populations. The third barrier (cc) was detected between FO-07 and FO-01.

### 2.4. Genetic Diversity Associated with Environmental Factors

**Figure 4:**
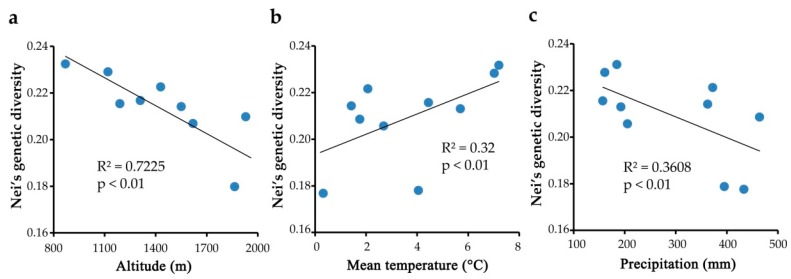
Regression analysis for Hj and environmental factors ((**a**) Hj and altitude; (**b**) Hj and mean temperature; (**c**) Hj and precipitation).

Pearson’s correlation analyses showed that the Nei’s genetic diversity (Hj) of *F. ovina* populations significantly decreased with increasing geographic altitude (*r* = −0.85, *p* < 0.01) (Table 5; Figure 4a), indicating a pattern of lower genetic diversity at higher altitudes. The population Hj was weakly positively correlated to annual mean temperature (*r* = 0.5657, *p* < 0.01) (Figure 4b), and weakly negatively related to annual precipitation (*r* = −0.6007, *p* < 0.01) (Figure 4c). While population Hj was not related to longitude or latitude. The Shannon diversity index (Ho) also presented a similarly significant pattern (*r* = −0.8368, *p* < 0.01) to altitude, and weak correlation to annual precipitation (*r* = −0.5715, *p* < 0.01). No significant correlation was found between Ho and annual mean temperature, longitude or latitude. The percent of polymorphic loci (PPL) was also weakly correlated to altitude (*r* = 0.6077, *p* < 0.01), and not related to annual mean temperature, annual precipitation, longitude or latitude.

## 3. Discussion

### 3.1. Population Genetic Diversity and Its Correlation to Environment Factors

Genetic diversity is considered the consequence of long-term evolution and represents the evolutionary potential of a species to survive in various environments [13,41]. Thus, a species must accumulate more genetic diversity in order to adapt itself to diverse environmental pressures [42]. AFLP with high levels of polymorphism represents a powerful tool for assessing genetic diversity in many species [37,43]. In this study, a total of six AFLP primer pairs generated 583 clear bands, of which 392 were polymorphic; thus, the PPB was 65.33%. The PIC ranged from 0.1917 to 0.2221, with an average of 0.2107. Previous research by Majidi et al. [44], in which the genetic variation of *Fescue* accessions was assessed using a DNA bulking strategy and AFLP, revealed an average NPB (number of polymorphic bands) of 41 and a PPB of 85.4%. Higher PIC values, such as 0.2715 (0.1534–0.3842) for genomic SSRs (simple sequence repeat) and 0.2224 (0.0760–0.4289) for EST-SSRs (expressed sequence tag-simple sequence repeat), than those of tall fescue were revealed by Tehrain et al. [45]. Generally, the population mean genetic diversity, genetic distance, and population size are positively correlated [46]. In most cases, widespread species tend to possess high genetic diversity. Conversely, the mean genetic distance (GD) in this study was 0.1094 (0.0802–0.1508), which corresponded to an average genetic diversity of Hj = 0.2006 (0.1686–0.2225). Majidi et al. reported higher values of GD = 0.55 (0.12–0.81) and Hj = 0.480 (0.273–0.611) [44]. A molecular diversity analysis was conducted by Fjellheim et al. on Norwegian meadow fescue (*Festuca pratensis* Huds.) populations and Nordic cultivars, which revealed a lower genetic diversity (Hj = 0.1412) [47]. Typically, cross-pollinated species maintain high intra-population variability relative to their inter-population variability [48]. The total genetic diversity (Hj = 0.2822) and mean genetic diversity (Hj = 0.2006) indicated the existence of 20.71% genetic differentiation among *F*. *ovina* populations, which corresponded well to the Shannon differentiation coefficient of G’st = 0.2512. The study by Majidi et al. [44] revealed higher levels of G’st = 0.337 and Fst = 0.355 among populations, whereas Fjellheim et al. [47] showed different differentiation patterns, such as Fst among seed populations (0.308), Fst among leaf populations (0.310) and Fst among cultivars (0.204). Currently, the high level of genetic diversity mirrors the high diversification and variation during long-term natural selection and evolutionary history, and genetic diversity benefits more easily from the broad geographical distribution range and high polyploidy for out-crossing species [24,45]. Conversely, the observations of this study suggested a relatively low genetic diversity of *F. ovina* populations. This low observed value of genetic diversity was possibly caused by different molecular markers or DNA strategies (individual DNAs or bulk DNAs). Alternatively, habitat fragmentation could be the explanation. Since the formerly panmictic population became fragmented, random genetic drift over time has led to both genetic differentiation among, and loss of heterozygosity within the fragmented population [49]. In the alpine area of Xinjiang, human overexploitation, overgrazing and farmland expansion have led to the increasingly patchy distribution and isolation of *F. ovina*, resulting in decreased fitness and reduced population size and thereby causing the observed low genetic diversity [27].

Evidence for lower genetic diversity at higher altitudes was found based on Pearson’s correlation analysis between geographic altitude and Nei’s genetic diversity (*r* = −0.85, *p* < 0.01) or Shannon diversity index (*r* = −0.8368, *p* < 0.01). For out-crossing species, wind, insects and animals are essential pollinators that guarantee gene flow within and among populations, these factors also determine the seed distribution of *F. ovina* [28]. Fewer insects and animals are found in alpine areas due to adverse environmental factors, including low temperatures, intense ultraviolet radiation and strong winds [50,51]. As a result, the plant population size gradually decreases, and deleterious erosion of genetic diversity occurs due to elevated levels of inbreeding and increased random genetic draft. Generally, ambient temperature could affect the growth and even alter the diversity of plants species, particularly in extremely low or high temperature. In this study, the Nei’s genetic diversity was weakly positively correlated to annual mean temperature (*r* = 0.5657, *p* < 0.01) (Figure 4b), and it partly revealed that low temperatures were harmful to plant genetic diversity when considering the glacial area. A previous study showed that glacial refugias in Tianshan Mountains and Altay area had an obvious effect on alpine plant species because migration and expansion normally occurred from these refugias [33]. Thus, the low observed value of genetic diversity of *F*. *ovina* in this study could be partly due to historic climate oscillation. For instance, suffering from the extremely low temperature, extinction occurred in mountain-top populations during glacial periods, whereas lowland populations survived due to the more tolerable temperatures [29,31]. Those alpine populations might have expanded from the few lowland individuals during the interglacial periods; therefore, less genetic diversity was observed. Furthermore, Nei’s genetic diversity or Shannon diversity index (*r* = 0.5715, *p* < 0.01) was weakly negatively correlated with annual precipitation (*r* = −0.6007, *p* < 0.01) (Figure 4c), partly indicating that excess rainfall was adverse for population genetic diversity. In Xinjiang, the wet season resulting from a temperate continental climate is concentrated in May and June every year [52,53]. So the continuous heavy rainstorms during the flowering period of *F. ovina* at May would disrupt the fertilization process and even reduce the population genetic diversity. Typically, the local microenvironment conditions would have great changes (such as temperature, intensity and duration of sunlight, climate, etc.) as the latitude and longitude increasing, which affect the distribution and development of plant species. Whereas the longitude and latitude didn’t impact on genetic diversity of *F. ovina* populations in this study, these were probably limited by the scarcity of specimens, and further research with more sampling sites would confirm it.

### 3.2. Genetic Structure and Gene Flow among Fragmented Populations under Genetic Barriers

The population genetic structure can reveal the interactions of various evolutionary processes, such as habitat fragmentation, population isolation, and gene flow. In the present study, the UPGMA tree, PCoA and STRUCTURE analyses revealed that the 10 *F. ovina* populations comprised two clusters, and FO-04 and FO-09 were isolated from adjacent populations (Figure 1 and Figure 2). Considering the relatively small distribution range of this species, the structural pattern of these populations was not related to their geographical distance, but instead, to their fragmentation. Most of the molecular variation was assigned to within-population variation (Fst = 79.29%) (Table 4), which resulted from the high ploidy level and out-breeding of *F. ovina.* The possession of several copies of the genome and the outcrossing mating system of this species would favor the maintenance of high levels of both intra-individual and intra-population genetic diversity [24]. On the other hand, as discussed above, a relatively recent extinction and replacement could also explain the low genetic divergence among populations, leading to a lack of accumulated differential mutations over a relatively short evolutionary time [54].

Gene flow (Nm) has an important impact on population genetic structure, evolutionary biology, conservation biology, and ecology [55]. High levels of gene flow can hinder intraspecific genetic drift and interspecific differentiation [56]. In this study, gene flow was Nm = 0.9571 among populations across all sampling sites, indicating the presence of some genetic barriers against gene flow. Three genetic barriers were directly detected and demonstrated in Figure 3. The first (aa) and second (bb) barriers isolated FO-04 and FO-09, respectively, from their adjacent populations. This result was congruent with the UPGMA tree, PCoA and STRUCTURE analyses. A further calculation was conducted for Fst and Nm at a smaller scale. The gene flow and genetic differentiation between FO-05, FO-06, FO-10 and FO-04 were Nm = 1.1713 and Fst = 0.1759, respectively. The gene flow and genetic differentiation between FO-03, FO-10 and FO-09 were Nm = 1.3282 and Fst = 0.1584, respectively. FO-04 and FO-09 were distributed in the alpine mountains with higher elevation, and thus, they had difficulty interacting with lowland populations. The explanation for this was the fragmentation induced by deserts and high mountains. Interestingly, FO-10 was distributed in the similar alpine area as FO-04 and FO-09, but low gene flow (Nm = 1.2372) and high genetic differentiation (Fst = 0.1681) were found between these populations. An analogous differential pattern was also detected between FO-07 and FO-01 (Fst = 0.091) in lowland according to the third genetic barrier (cc). The reason for these might be the arid process and fragmentation due to desert expansion and high mountains that promoted the differentiation between adjacent populations [32,33]. Alternatively, FO-10 might be the colonization from lowland populations (as discussed above for historical climate oscillation), thereby explaining its similar genetic lineages and co-clustering in Cluster I (Figure 1).

## 4. Materials and Methods

### 4.1. Plant Materials and Genomic DNA Preparation

**Figure 5:**
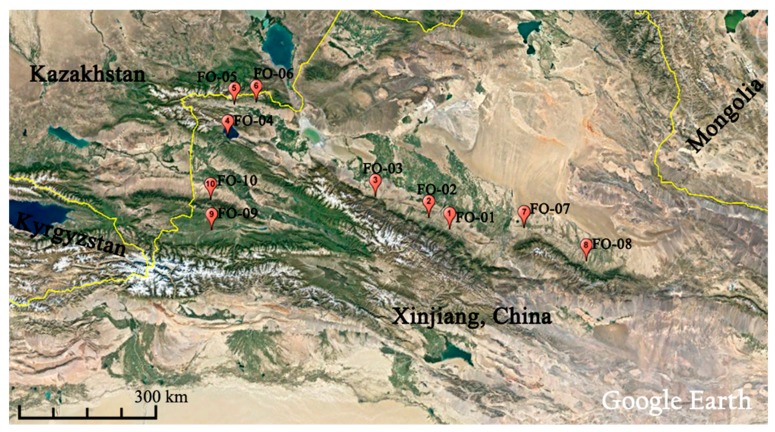
Geographical locations of analyzed populations of *Festuca ovina* in Xinjiang, China.

A total of 10 wild *F. ovina* populations (10 individuals per population) were sampled from Xinjiang, China in 2010 (Table 6). Individual spikes over 25 m apart from one another were sampled randomly to assure that they were from different individuals (Figure 5). A single seed from each collected single spike was germinated at Sichuan Agricultural University using an individual pot in an illumination incubator at an approximate temperature of 22 °C and under a 16 h photoperiod. Before DNA extraction, chromosome identification was carried out using the squashed root tips technique [57] for each individual 3-day-old germinated seedling to guarantee the DNA purity. The results suggested that all individuals and populations were diploid (2*n* = 2*x* = 14).

For each individual, genomic DNA was extracted from an aggregate of 3–5 fresh leaf tissues (approximately 80 mg) using a plant DNA extraction kit (Tiangen, Beijing, China) according to the manufacturer’s instructions. The DNA concentration was quantified using a NanoDrop^®^ ND-1000 Spectrophotometer (NanoDrop Technologies, Wilmington, DE, USA) and diluted to 100 ng/μL for AFLP analysis.

### 4.2. AFLP Procedure

The AFLP procedure was performed according to Sorkheh et al. [43]. A preliminary amplification using a set of 100 primer pairs identified six selective primer pairs as the most informative and reliable; these six primers were used for amplification. The PCR amplification reaction was performed in a 20 μL system: 1 PCR buffer, 2 mM MgCl_2_, 2 mM dNTP, 40 ng of each of *Eco*RI primer and *Mse*I primer, 1 U *Taq* polymerase, and 5.0 µL pre-amplified template DNA. The fragments amplified in the latter step were subjected to capillary electrophoresis using an ABI 3500 (Applied Biosystems, Foster City, CA, USA). GeneMarker (version 2.6, SOFTGENETICS, State College, PA, USA) was used to treat the fluorescent AFLP fragments for each individual sample.

### 4.3. Data Analysis

#### 4.3.1. Genetic Diversity

Each band in the AFLP fingerprint pattern was considered a separate putative locus, and the bands clearly indicating AFLP amplification as 1 (presence) or 0 (absence) in a readable range (between 50 and 400 bp) were used to generated the binary matrices. The following parameters were calculated using EXCEL 2013 software (Microsoft, Redmond, WA, USA): total number of bands (TNB), number of polymorphic bands (NPB), percentage of polymorphic bands (PPB) and polymorphism information content (PIC, according to Zhang et al. [58]). The Shannon information index (Ho), observed number of alleles per locus (Na), and effective number of alleles per locus (Ne) were calculated using POPGENE (version 3.0, University of Alberta, Edmonton, AB, Canada) [59] under Hardy–Weinberg equilibrium (HWE). Nei’s genetic diversity (Hj) was calculated using AFLP-SURV (version 1.0, ULB, Belgium) [60].

#### 4.3.2. Genetic Structure

First, Nei’s genetic distance (GD) of the population was calculated using AFLP-SURV v1.0 [60] with 10,000 bootstraps, and the results were used as inputs for computing the unweighted group method with arithmetic mean (UPGMA) tree using the CONSENSE module in PHYLIP (version 3.69, University of Washington, Seattle, WA, USA) [61]. Second, principal coordinates analysis (PCoA) for 100 individuals was conducted using the R packages “stats” [62] and “scatterplot3d” [63] to evaluate their spatial distribution pattern. Third, Bayesian model-based cluster analysis was performed to infer the number of clusters using the software STRUCTURE (version 2.3.4, Pritchard Lab, Stanford University, Stanford, CA, USA) [64] with correlated allele frequencies and an admixed model with a burn-in period of 50,000 and 200,000 MCMC (Monte carlo in markov chain) replicates after burn-in. The predefined cluster (K) was 1–10 with 10 runs per K. The optimum K was decided by determining L(K) and ΔK (the subtraction of two continuous L(K) values) using STRUCTURE HARVESTER (version 0.6.94, UCSC, Santa Cruz, CA, USA) [65]. Those values usually reached a plateau or increased slightly after reaching the “optimum K”.

#### 4.3.3 Genetic Variation, Gene Flow and Genetic Barrier

To further study the genetic differentiation (Fst) within and among populations, analysis of molecular variance (AMOVA) was conducted at different levels using the R package “vegan” [66]. The Shannon differentiation coefficient (G’st) was calculated according to the following formula: G’st = (Hsp − Hpop)/Hsp (Hsp, total Shannon information index; Hpop, average Shannon information index within the population). Gene flow (Nm) was calculated as Fst (Nm = (1 − Fst)/4Fst). Moreover, a genetic barrier analysis was devised to suggest historical barriers to gene flow among or between collection sites using software BARRIER (version 2.2, Syracuse University, USA) [67] according to Monmonier’s maximum difference algorithm, which treated geographical coordinates and genetic distance (GD) of each population as inputs.

#### 4.3.4. Correlation Analysis

To examine the level of significance, the Pearson correlation between genetic diversity and environmental factors (annual mean temperature, annual precipitation, longitude, latitude and elevation) was estimated using the R package “ade4” (version 1.7.6, Aurélie Siberchicot) [68] with 1000 random permutations.

## 5. Conclusions

The results of this study demonstrate that environmental conditions in different habitats are major factors influencing the hereditary characteristic of species. The genetic diversity of *F. ovina* was relatively low. Population genetic diversity was decreased with increasing elevation, and it was also weakly related to temperature and precipitation but not correlated to latitude or longitude. Restricted gene flow was observed at different spatial scales, whereas genetic differentiation due to fragmentation was also detected between adjacent populations.

## Figures and Tables

**Table 1:** Summary of genetic diversity based on AFLP loci amplified by each primer combination. Total number of bands (TNB), number of polymorphic bands (NPB), percentage of polymorphic bands (PPB), polymorphism information content (PIC), Nei’s genetic diversity (Hj), Shannon diversity index (Ho).

Primer	TNB	NPB	PPB (%)	PIC	Hj	Ho
E42M57	109	70	64.22	0.2132	0.2538	0.2136
E42M85	89	56	62.92	0.2221	0.3024	0.3617
E85M57	101	66	65.35	0.1917	0.2628	0.4406
E85M85	103	68	66.02	0.2152	0.2464	0.2261
E86M57	90	67	74.44	0.2095	0.2036	0.2046
E86M85	91	65	71.43	0.2124	0.2499	0.4116
Total	583	392	67.24	0.2107	0.3549	0.4642
Mean	97.17	65.33	67.24	0.2107	0.2531	0.3164

**Table 2:** Genetic variability in 10 *Festuca ovina* populations. Number of polymorphic loci (Np), percentage of polymorphic loci (PPL), observed number of alleles per locus (Na), effective number of alleles per locus (Ne), Nei’s gene diversity index (Hj), Shannon diversity index (Ho).

Population	Np	PPL (%)	Na	Ne	Hj	Ho
FO-01	272	69.4	1.6582	1.2982	0.2042	0.2343
FO-02	271	69.1	1.6709	1.3401	0.2191	0.2469
FO-03	282	71.9	1.6607	1.2920	0.1969	0.2214
FO-04	198	50.5	1.4719	1.2625	0.1686	0.1966
FO-05	274	69.9	1.6786	1.3227	0.2126	0.2461
FO-06	294	75.0	1.7092	1.3006	0.2054	0.2327
FO-07	271	69.1	1.6735	1.3425	0.2225	0.2505
FO-08	256	65.3	1.6352	1.3140	0.2068	0.2336
FO-09	202	51.5	1.5026	1.2585	0.1698	0.1982
FO-10	273	69.6	1.6531	1.3119	0.1998	0.2264
Total	392	100	2.0000	1.3571	0.2622	0.2988
Mean	298.5	67.24	1.6314	1.3043	0.2006	0.2287

**Table 3:** Nei’s genetic distance matrix of 10 *F. ovina* populations based on AFLP (amplified fragment-length polymorphism) profiles. Genetic distance (GD).

GD	FO-02	FO-03	FO-04	FO-05	FO-06	FO-07	FO-08	FO-09	FO-10
FO-01	0.0823								
FO-02	0.0853	0.0804							
FO-03	0.1504	0.1418	0.1504						
FO-04	0.0866	0.0802	0.0890	0.1432					
FO-05	0.088	0.0833	0.0811	0.1477	0.0814				
FO-06	0.0867	0.0869	0.0921	0.1486	0.0919	0.0942			
FO-07	0.0934	0.0957	0.0978	0.1473	0.0952	0.0999	0.0889		
FO-08	0.1486	0.1393	0.1499	0.0911	0.1441	0.1462	0.1483	0.1495	
FO-09	0.0897	0.0829	0.0836	0.1508	0.0824	0.0839	0.0962	0.0986	0.1498

**Table 4:** Analysis of molecular variance for *F. ovina* populations.

Group	Source of Variation	D.f.	Sum of Squares	Variance Components	Percentage of Variation	F-Statistic	*p*-Value
Two clusters	Among clusters	2	2334.77	11.98	11.54	Fct = 0.1154	<0.01
	Among pops. within clusters	7	2143.16	9.74	10.54	Fsc = 0.1054	<0.01
	Within populations	89	4477.93	46.53	77.92	Fst = 0.2208	<0.01
	Total	98	5647.54	58.68			
All pops.	Among populations	9	1169.60	12.15	20.71%	Fst = 0.2071	<0.01
	Within populations	89	4477.93	46.53	79.29%		
	Total	98	5647.54	58.68			

**Table 5:** Pearson correlation analysis (*r*) between genetic diversity and environmental factors.

Variable	Pearson Coefficient	Altitude	Annual Mean Temperature	Annual Precipitation	Longitude	Latitude
Hj	*r*	−0.8500	0.5657	−0.6007	0.5291	0.2953
	*p*	0.0024	0.0096	0.0017	0.0024	0.0235
Ho	*r*	−0.8368	0.5433	−0.5715	0.5056	0.3288
	*p*	0.0076	0.0120	0.0089	0.0032	0.0233
PPL	*r*	−0.6077	0.2022	−0.3919	0.2720	0.3975
	*p*	0.0033	0.1310	0.0185	0.0118	0.0131

**Table 6:** List of the 10 wild *F. ovina* populations in this study.

Population	Altitude (masl)	Annual Mean Temperature (AMT, °C)	Annual Precipitation (AP, mm)	Longitude	Latitude	Grassland Type	Habitat and Dominant Herbs
FO-01	1550	5.7125	207	86°22′0″	43°53′1″	Temperate desert steppe	Ungrazed slope above road, with Leymus, Koeleria and Stipa
FO-02	1120	7.0583	175	85°52′13″	44°0′23″	Temperate desert steppe	Fenced pasture, with Artemisia and Seriphidium
FO-03	1620	2.6625	220	84°38′5″	44°9′12″	Temperate desert steppe	Natural pasture, with Stipa, Seriphidium, Aster and Festuca
FO-04	2065	0.1375	451	81°8′24″	44°31′0″	Temperate meadow steppe	Lakeside, with Stipa, Carex, Festuca, Taraxacum and Potentilla
FO-05	1430	2.0333	389	81°5′8″	45°3′7″	Temperate steppe	Heavily grazed hill, with Artemisia, Potentilla and Seriphidium
FO-06	1190	1.3750	379	81°33′9″	45°10′0″	Temperate steppe	Heavily grazed hill, with Artemisia, Potentilla and Seriphidium
FO-07	870	7.2417	199	87°58′17″	44°7′22″	Temperate desert steppe	Non-irriaged mountain pasture, with Stipa, Seriphidium and Aster
FO-08	1310	4.4417	171	89°27′32″	43°46′0″	Temperate desert steppe	Dry hills used for winter pastures, with Stipa, Seriphidium and Aster
FO-09	1880	4.0458	412	81°18′23″	43°1′42″	Temperate meadow steppe	Ungrazed hillside, with Stipa, Carex, Festuca and Taraxacum
FO-10	1960	1.7125	482	81°7′24″	43°29′26″	Temperate meadow steppe	Moderately grazed hillside, with Stipa, Carex, Taraxacum and Oxytropis
